# Degree of control of patients with chronic obstructive pulmonary disease in Spain: SINCON study

**DOI:** 10.1186/s12890-018-0749-7

**Published:** 2018-12-03

**Authors:** Adolfo Baloira, José Miguel Rodriguez Gonzalez-Moro, Estefanía Sanjuán, Juan Antonio Trigueros, Ricard Casamor

**Affiliations:** 1 Hospital de Montecelo, Mourente, s/n, 36071 Pontevedra, Spain; 2 Hospital Universitario Príncipe de Asturias, Alcalá de Henares, Madrid, Spain; 3 CAP María Bernades, Viladecans, Barcelona, Spain; 4 Centro de Salud Menasalbas, Toledo, Spain; 5 Novartis Farmacéutica S.A, Barcelona, Spain

**Keywords:** COPD, Disease control, CAT, mMRC, Exacerbations

## Abstract

**Background:**

Disease control is an important objective of COPD management. The SINCON study evaluated the level of control in terms of respiratory symptoms and exacerbations in Spanish patients with COPD for ≥2 years.

**Methods:**

SINCON was a descriptive, cross-sectional, multicenter study that assessed degree of control using a combined index comprising COPD assessment test (CAT), modified Medical Research Council dyspnea scale (mMRC), and number of moderate/severe exacerbations in the last year. Based on this score, patients were categorized as “well controlled” or “poorly controlled”. Degree of control was also assessed relative to patient phenotype, setting (primary care [PC] vs respiratory care [RC]), and impact of treatment on morning symptoms.

**Results:**

Of the 481 patients (PC: 307, RC: 174) analyzed, COPD was poorly controlled in 63.2%. Some differences were found between clinical settings: PC patients were more poorly controlled (PC: 66.4% vs RC: 57.5%; *P* = 0.06) and had higher CAT score (PC: 17.9 vs RC: 15.5; *P* < 0.05), and higher rate of moderate/severe exacerbations during previous year (PC: 1.5 vs RC: 1.1; *P* < 0.05), while dyspnea degree was similar in both settings. Regarding phenotypes, non-exacerbators demonstrated better control vs exacerbators. Morning symptoms score improved between waking and 3 h after bronchodilator treatment (*P* < 0.05), with greater improvements in PC patients (PC: − 6.5 vs RC: − 5.0 points; *P* < 0.05).

**Conclusions:**

Most COPD patients were poorly controlled with some differences observed between PC and RC settings and between patient phenotypes. Our index may be easily used in PC settings to optimize COPD treatment.

## Background

Chronic obstructive pulmonary disease (COPD) is usually characterized as having a progressive clinical course, interrupted by more or less frequent exacerbations over time. The speed of lung function loss may vary greatly and seem mildly impacted by the available treatments, as observed in the UPLIFT study [1]. The key objectives of treatment are to decrease symptoms, improve quality of life (QoL) and prevent exacerbations and, therefore, the definition of control of the disease should include these clinical variables. COPD Assessment Test (CAT) is a simple questionnaire developed to know how the disease affects the patient’s life. CAT has been validated in different studies, being one the most important tools for the GOLD strategy [2–4]. Due to its simplicity, it can be easily used in the daily clinic. Dyspnea is possibly the most important symptom in patients with COPD. The modified Medical Research Council (mMRC) scale is an easy way to know the limitations that the patient presents due to dyspnea. It is included in the GOLD guidelines along with the CAT questionnaire, but both are not equivalent [5]. It is widely used in clinical trials and correlates with different clinical variables in COPD, being a better predictor of mortality than FEV1 [6]. In general, its use is recommended in the follow-up of patients.

Bronchodilators are the cornerstone in the long-term treatment of stable COPD, as recognized in the various guidelines and recommendations [7], while inhaled corticosteroids (ICS) may also play a relevant role in certain patients [8]. Given the progressive nature of the disease, the therapeutic strategy should be a progressive combination of drugs, depending on clinical variables (symptoms, exacerbations, and lung function), but adequate measuring tools are not always well established. The objective should be to achieve a good degree of disease control. However, it is not easy to define control in a specific patient with a disease that is as variable in its clinical manifestations as COPD. The term “control” (well-controlled or poorly controlled patient) should refer to both the clinical situation at a certain time and the risk of worsening or death in the relatively near future [9]. Since COPD has various levels of severity, the patient’s degree of control will be greatly influenced by the level of severity, since, unlike asthma, it is not possible to fully reverse bronchial obstruction or the underlying inflammation.

A key aspect of control is to define the clinical phenotype of the patient. Guía Española de la EPOC (GesEPOC) was the first guideline to classify COPD per clinical phenotypes and recommend treatments accordingly [10]. Exacerbations are a crucial aspect of COPD progression, and influence QoL, lung function loss and mortality risk [11, 12]. Several studies have shown a positive effect of different available treatments on reduction of exacerbations [13–15]. In light of the current knowledge, clinical guidelines have created simple therapeutic algorithms for easy application in clinical practice, which should allow for better control of patients with COPD. Using a British database, an international group tried different approaches to evaluate the control of patients, using either clinical aspects or the CAT questionnaire, with different thresholds. The results were different, as expected, depending on the criteria used, but in all cases, worse control was associated with a greater probability of having an exacerbation [16]. More recently, the same group has conducted a study with the aim to validate this concept of control. This study found that an exacerbation in the three previous months and a high score in questionnaires of dyspnea and quality of life were associated to classify the patient as poorly controlled [17].

There is little information on the degree of control of patients with COPD, applying easy-to-use indexes in daily clinical practice. The main objective of the SINCON study was to assess the degree of control of COPD patients in Spain, taking into account the symptoms and exacerbations, the most important variables associated with the concept of control according to available data. As secondary objectives, we assessed the degree of control related to patient phenotype or level of care (primary care [PC] physicians vs respiratory care [RC] specialists) and the impact of treatment on morning symptoms.

## Methods

SINCON was a descriptive, cross-sectional, multicenter study with representation from all the Spanish geography. In order to obtain a representative sample of the Spanish population, the number of patients was limited to a maximum of 5 for each PC doctor and 10 in the case of RC. Obviously, PC could not include patients who were also followed by pulmonologists This study was conducted in accordance with the ICH Harmonised Tripartite Guidelines for Good Clinical Practice, with applicable local regulations (including the European Directive 2001/83/EC and U.S. Code of Federal Regulations Part 21), and with the ethical principles established in the Declaration of Helsinki. The study protocol, including de informed consent, was reviewed and approved by the Institutional Review Board of Complejo Hospitalario de Toledo (23 December 2014, protocol number 156). All patients signed the informed consent before being included in the study. To minimize screening bias, patients were recruited consecutively from among those who attended the outpatient clinic, met the inclusion and exclusion criteria, and signed the informed consent. Inclusion criteria were age > 40 years, COPD diagnosis for ≥2 years (post-bronchodilator forced expiratory volume in 1 s [FEV_1_] < 80% of the theoretical value and FEV_1_/forced vital capacity [FVC] ratio < 0.7 measured in scheduled visit), no exacerbations in the last 3 months, able to follow the study protocol and willing to sign the informed consent. Patients with symptomatic systemic diseases, respiratory conditions other than COPD, serious diseases with a life expectancy of < 1 year, or unable to respond to the questionnaires were excluded.

The primary objective of the study was to assess the degree of control of COPD. For this purpose, we created a combined index that included three variables: COPD Assessment Test (CAT), modified Medical Research Council dyspnea scale (mMRC) and number of moderate/severe exacerbations in the previous year. An exacerbation was considered as moderate when it required treatment with systemic corticosteroids and/or antibiotics and as severe when the patient visited a hospital, with emergency room stay of at least 12 h or hospital admission. The assignment of scores to each of these variables is described in Table 1. The degree of control was defined by the sum of scores, establishing the four groups: optimal control (0 points), suboptimal control (1 point), poor control (2 points), and very poor control (3 points). To better summarize the outcomes, patients with optimal or suboptimal control were considered “well controlled” and patients with poor and very poor control were considered “poorly controlled”.

**Table 1:** Assignment of scores to individual clinical variables

Clinical Variable	Value	Score
mMRC	0,1	0
	2,3,4	1
CAT	< 10	0
	≥ 10	1
Exacerbations in the previous year	none or one with no hospitalization	0
	more than one or one with hospitalization	1

*CAT* COPD assessment test, *mMRC* modified Medical Research Council dyspnea scale

In order to assess morning symptoms and the impact of treatment on these symptoms, a patient-reported outcome questionnaire previously validated was used [18]. Patients completed the first part when they woke up at home and the second part, 3 h after the administration of bronchodilator medication, at the doctor’s office. The score for each part varied between 0 and 60; the higher the score, the higher the severity of the morning symptoms. The following variables were also included: age, sex, body mass index, smoking status, comorbidities, time since COPD diagnosis, phenotype as per GesEPOC guidelines (assessed by local investigator), lung function with post-bronchodilator spirometry, treatment for the last 3 months and history of exacerbations in the previous year. All data were collected in a single visit.

### Statistical analysis

A sample size of 700 patients was calculated, which corresponded to 0.14% of the total COPD population diagnosed in Spain for a margin of error of ±3.70% [19]. Continuous variables were expressed as mean and 95% confidence interval (CI), and qualitative variables as absolute and relative frequency. Differences in the degree of control per phenotype were analyzed using the chi-square or Fisher’s exact test depending on the sample. The significance level was set to *P* < 0.05.

## Results

**Fig. 1:**
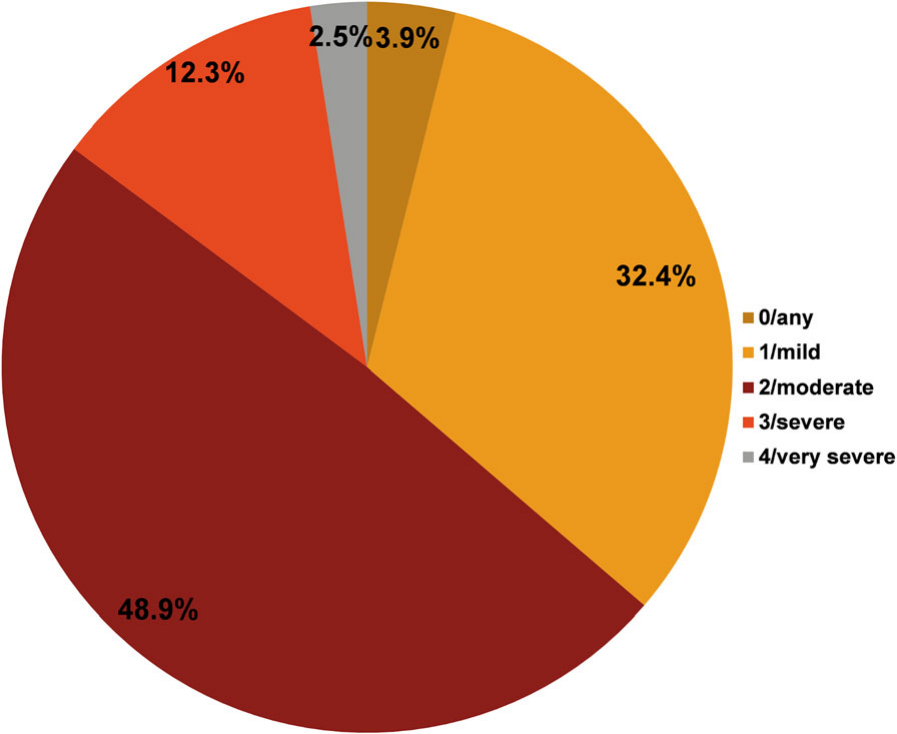
Percentage of patients according to mMRC scale mMRC, modified Medical Research Council dyspnea scale

**Fig. 2:**
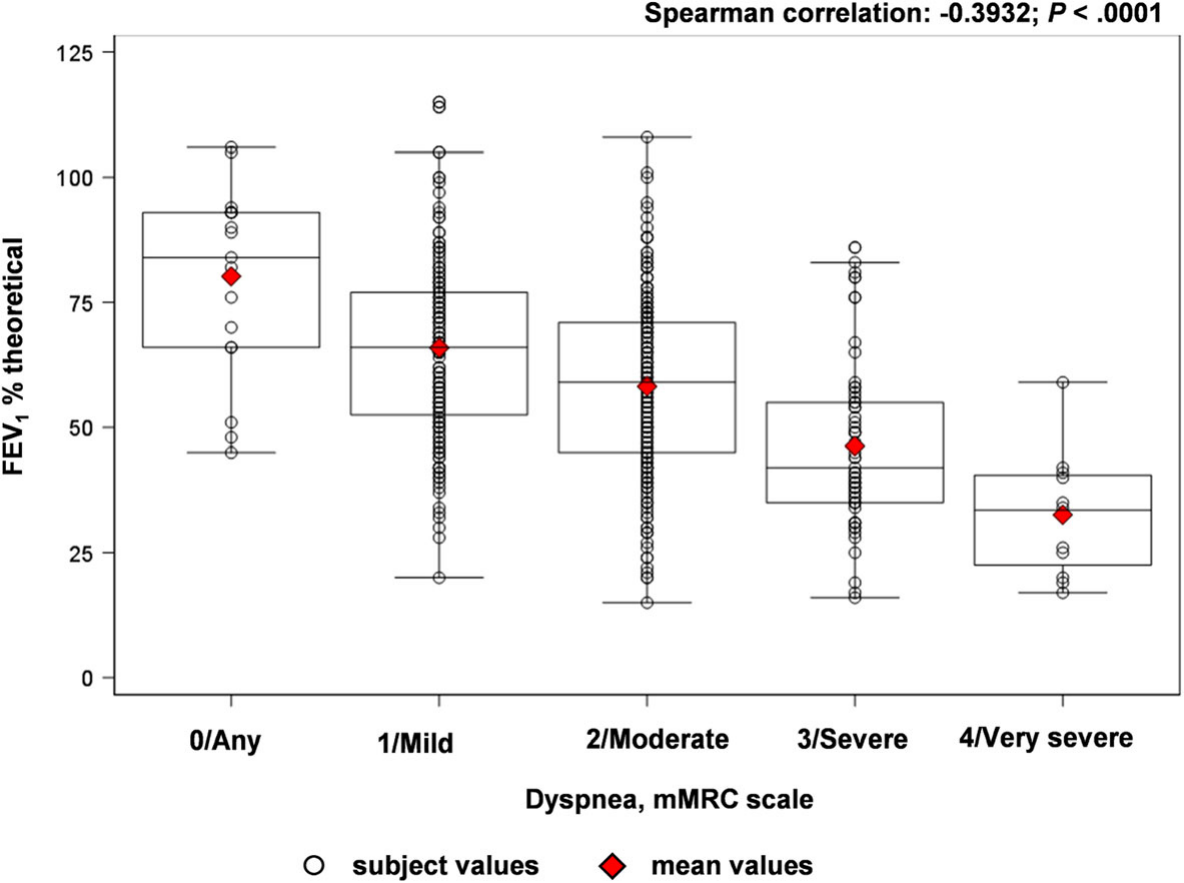
Correlation between pulmonary function (FEV_1_) and dyspnea FEV_1_, forced expiratory volume in 1 s; mMRC, modified Medical Research Council dyspnea scale

**Fig. 3:**
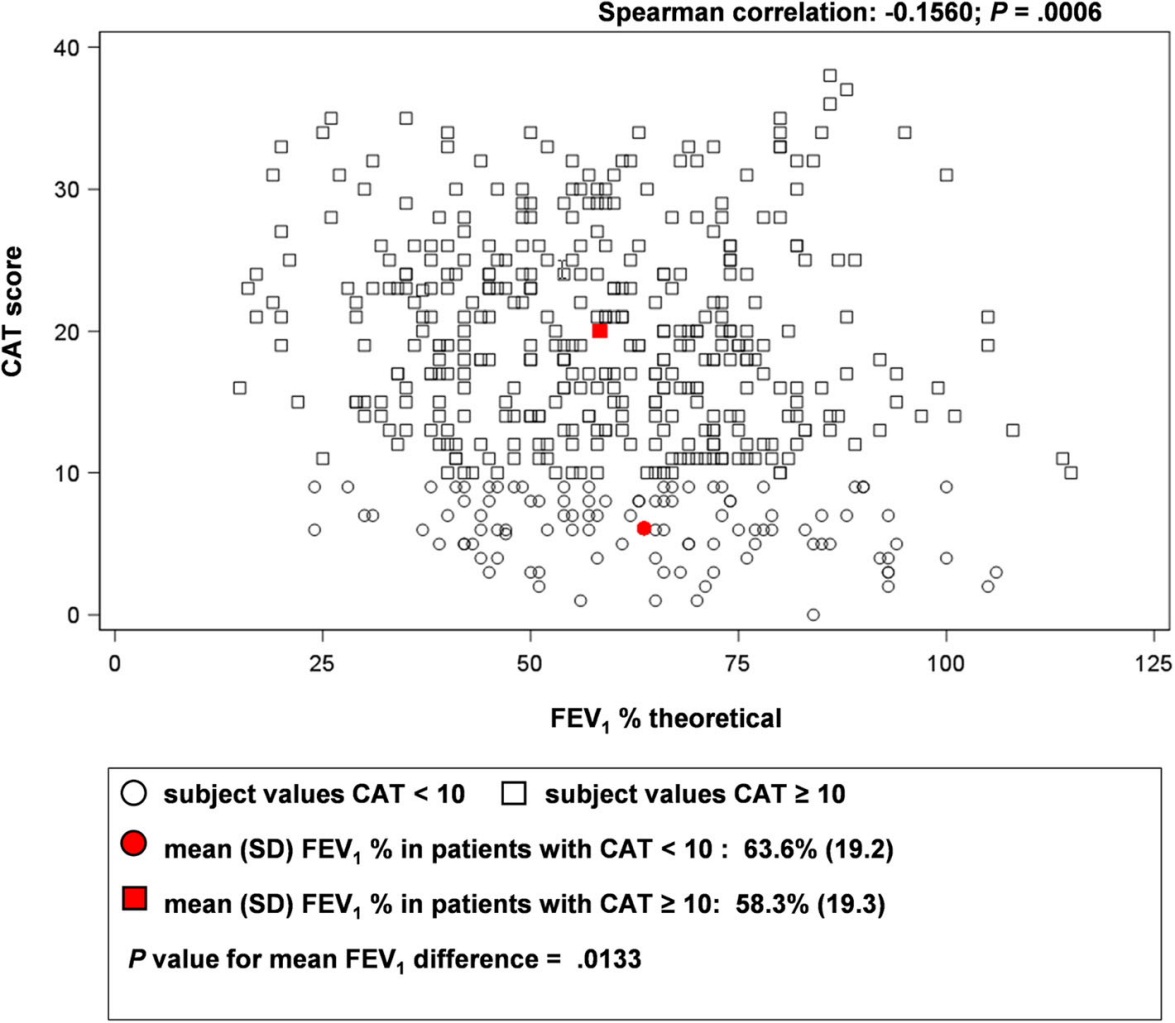
Correlation between CAT and pulmonary function (FEV_1_) CAT, COPD assessment test; FEV_1_, forced expiratory volume in 1 s; SD, standard deviation

A total of 100 PC centers and 33 RC departments participated. Initially, 584 patients were enrolled; 103 patients were excluded as they did not satisfy inclusion/exclusion criteria or did not complete the information required. The analysis sample comprised 481 patients (18.5% female) with a mean (± SD) age of 67 (± 10) years (Table 2). Of the total patients analyzed, 307 and 174 patients were monitored in PC and RC settings, respectively. The mean (± SD) exposure to tobacco was 39.8 (± 22.4) pack-years and 62.8% of patients were former smokers. The mean (± SD) time since COPD diagnosis to the visit was 9.3 (± 7.1) years. The mean (± SD) FEV_1_ was 59% (± 19%) of the theoretical value, and the mean (± SD) FEV_1_/FVC ratio was 0.58 (± 0.14). The majority of patients had dyspnea grade 2–4 (moderate-to-very severe) (Fig. 1), both in PC and RC settings. The mean CAT score was 17.0 (95% CI, 16.3–17.8), which was slightly lower in patients from RC departments (PC: 17.9 vs RC: 15.0; *P* < 0.05). A significant correlation was observed between FEV_1_ and dyspnea grade (*P* < 0.0001) (Fig. 2), between dyspnea and CAT score (*P* < 0.0001) and between FEV_1_ and CAT score (*P* = 0.0133) (Fig. 3).

**Table 2:** Characteristics of patients

Characteristic	Evaluable COPD patients (*N = 481*)
Sex, male, n (%)	392 (81.5)
Age, years, mean (SD)	67.7 ± 10.0
Body mass index, kg/m^2^	27.9 ± 4.6
FEV_1_, % theoretical	59.4 ± 19.4
FEV_1_/FVC ratio	0.6 ± 0.2
Current smokers, n (%)	179 (37.2)
Former smokers, n (%)	302 (62.8)
Tobacco exposure, pack-years	39.8 ± 22.4
Time since COPD diagnosis, years	9.3 ± 7.1

Data are presented as mean ± SD unless specified otherwise

*COPD* Chronic obstructive pulmonary disease, *FEV*_*1*_ forced expiratory volume in 1 s, *FVC* forced vital capacity, *SD* standard deviation

In terms of exacerbations, 58.6% of patients had at least one moderate or severe exacerbation during the previous year (Table 3). PC patients showed a significantly higher frequency of moderate exacerbations per patient during the previous year, compared with RC patients (PC: 1.3 vs RC: 0.8; *P* < 0.05). The rate of severe exacerbations was low, with a tendency to be more frequent in RC patients, although the difference did not achieve statistical significance (PC: 0.2 vs RC: 0.3; *P* = 0.91). Based on clinical phenotypes, 46% of patients were classified as non-exacerbators, 13.7% as exacerbators with emphysema, 28.3% as exacerbators with chronic bronchitis, and 10.6% as asthma-COPD overlap phenotype. In seven cases, the phenotype could not be established.

**Table 3:** Recorded Exacerbations in Previous Year

	PC (*N = 307*)	RC (*N = 174*)	Total (*N = 481*)
Moderate exacerbations, *n* (%)	188 (61.2%)	82 (47.1%)	270 (56.1%)
Rate^a^, mean (95% CI)	1.3 (1.1–1.5)	0.8 (0.6–0.9)	1.1 (1.0–1.2)
Severe exacerbations, *n* (%)	39 (12.7%)	33 (18.9%)	72 (14.9%)
Rate^a^, mean (95% CI)	0.2 (0.1–0.2)	0.3 (0.2–0.4)	0.2 (0.2–0.3)
Moderate/Severe exacerbations, *n* (%)	191 (62.2%)	91 (52.3%)	282 (58.6%)
Rate^a^, mean (95% CI)	1.5 (1.3–1.7)	1.1 (0.8–1.3)	1.3 (1.2–1.5)

^a^events/patient/year

*CI* confidence interval, *PC* primary care, *RC* respiratory care

Analysis of the degree of control showed that 14 and 23% of patients achieved optimal and suboptimal control, respectively, with the remaining 63% of patients being poorly controlled, in accordance with the definition proposed (Table 4). A slightly higher proportion of patients in PC vs RC were poorly controlled, though this difference was not statistically significant (PC: 66.5% vs RC: 57.5%; *P* = 0.06). Significant differences were observed depending on clinical phenotype: non-exacerbating patients showed poor control in 41.6% of cases, while a higher percentage of exacerbating patients with chronic bronchitis (86.0%) and emphysema (87.9%) had poor control (non-exacerbating vs exacerbating; *P* < 0.0001). Patients in good control received more often dual bronchodilation than a long-acting β_2_-agonist (LABA)/ICS combination (good control percentage of treatments, DBD: 72.5% vs LABA/ICS: 45.0%; *P* < 0.05). Patients with bad control were treated with ICS more frequently in RC than in PC (63,2% vs 31.5% respectively, *P* < 0.01).

**Table 4:** Degree of Control of COPD Patients

Degree	PC (*N = 307*)	RC (*N = 174*)	Total (*N = 481*)
0: Optimal, n (%)	32 (10.4)	33 (18.9)	65 (13.5)
1: Suboptimal, n (%)	71 (23.1)	41 (23.5)	112 (23.2)
2: Poor, n (%)	106 (34.5)	58 (33.3)	164 (34.1)
3: Very Poor, n (%)	98 (31.9)	42 (24.1)	140 (29.1)

*COPD* chronic obstructive pulmonary disease, *PC* primary care, *RC* respiratory care

Although there was a trend to a better degree of control in RC, the differences did not reach statistical significance

In terms of morning symptoms and changes after bronchodilator treatment (Table 5), the mean score in the patient-reported outcome questionnaire decreased significantly (indicating symptom improvement) between waking and the evaluation performed after 3 h at the clinic (mean improvement: − 6.5; 95% CI, − 7.1– − 4.1 points; *P* < 0.05). In patients from PC this improvement was − 7.3 (− 8.2– − 6.5) points, while in patients from RC it was − 5.0 (95% CI, − 5.9– − 4.1) points. This improvement occurred regardless of GOLD stage. Patients in stages B and D, most symptomatic, obviously had a worse score in the PRO questionnaire. Patients treated with LABA/LAMA showed a greater improvement than those treated with a LABA/ICS or with a long-acting muscarinic antagonist (LAMA) alone (LABA/LAMA: − 7.4; 95% CI, − 8.4– − 6.5 vs LABA/ICS: − 6.2; 95% CI, − 7.3– − 5.0 vs LAMA: − 5.5; 95% CI, − 6.4– − 4.6) but without reaching statistical significance (*p* = 0.06) (Table 6).

**Table 5:** PRO questionnaire according to 2015 and 2017 GOLD stage

	GOLD	Patients *N* 475	Waking (SD)	3 h after treatment (SD)
GOLD 2015	A	57	6.61 (9.44)	4.21 (8,68)
	B	154	18.46 (12.81)	11.46 (9.83)
	C	25	5.84 (5.20)	3.34 (3.19)
	D	239	21.51 (13.37)	13.61 (10.25)
GOLD 20017	A	70	6.47 (8.73)	4.18 (7.91)
	B	225	18.27 (12.45)	11.56 (9.53)
	C	12	5.83 (5.92)	2.33 (3.36)
	D	168	23.05 (13.75)	14.52 (10.66)

Significant differences (*p* < 0.01) in all cases before and after treatment. There were no differences between GOLD 2015 and GOLD 2017

**Table 6:** PRO questionnaire according to treatment

Long-acting COPD treatment	Waking	3 h after treatment	Difference between both evaluations
LABA/LAMA	19.9 (18.8, 21.8)	12.3 (10.9,13.7)	−7.4 (− 8.4,-6.5)
LABA/ICS	18.0 (15.8, 20.1)	11.9 (10.1,13.6)	−6.2 (−7.3,-5.0)
LAMA	16.6 (14.7,18.5)	11.1 (9.6,12.7)	−5.5 (−6.4,-4.6)

All values mean (95% CI). Difference between LABA / LAMA and LAMA with a trend to statistical significance (p = 0.06)

## Discussion

The concept of control in a disease such as COPD is difficult to define and quantify, as there is usually a progressive lung function loss that seems to be only mildly modified by current treatments. In this study, we used three variables that constitute the central axis of the clinical manifestations of COPD: dyspnea, QoL and exacerbations. Considering the impact of these variables on patients, achieving optimal control seems to be a major challenge. Some factors that appear to influence the proportion of patients who achieve good control include phenotype, lung function and, to a lesser degree, the level of care in the setting where the patient is seen (PC vs RC).

### Concept of control

In the absence of a well-established definition for COPD control, we developed and used an index that included some of the parameters most related to the impact of the disease, based both on the symptoms perceived by the patient and on the prognosis. Lung function has traditionally been a key aspect in the management of COPD patients; however, it has some drawbacks.

1. The references used to measure the lung function allow some differences that, in many cases, exceed the efficacy outcomes obtained in clinical trials [20]
2. Lung function has significant intra-patient variations, thus an isolated value may be mildly significant
3. Even though there is a certain correlation between lung function and dyspnea or QoL, there is a significant dispersion of results
4. It has not yet been proven that the available drugs slow the loss of lung function. Therefore, it is difficult to justify lung function as a parameter when trying to assess the degree of control of COPD patients. It is obviously more difficult to achieve an adequate control in patients with severely impaired lung function than in those with mild obstruction. However, since the primary objective of the study was to obtain a global view of the degree of control in a set of patients from Spain, we chose not to include lung function in our index.

### Quality of life and dyspnea: Do we have appropriate tools to measure them?

Quality of life and symptoms are two key aspects when designing a definition of control in patients with COPD. Due to their subjective nature, they are difficult to measure. The mMRC dyspnea scale and the CAT questionnaire are two semi-quantitative tools used in clinical trials and in clinical practice. They have proven to be effective in terms of prognosis and are sensitive to the effect of different drugs used in COPD treatment [21]. The Global Initiative for Chronic Obstructive Lung Disease (GOLD) strategy document recommends either of these tools to assess the patients’ symptoms; however, these tools actually explore different aspects of the disease and their degree of consistency varies greatly [22]. CAT has an acceptable correlation with the GOLD classification [23]. It is difficult to determine a strict cut-off point to define a good/poor score, since in reality these scales or questionnaires are inaccurate in their limits and rather work as a continuum. Therefore, it seemed appropriate to use both when creating an index to assess the patients’ control. Good control was defined in our index considering the cut-off values that GOLD recommends to classify a low-symptom patient, both for the mMRC scale and the CAT questionnaire, but obviously this is debatable.

### Controversial aspects in exacerbations

Exacerbations are another key aspect of COPD progression with a significant impact on the patient’s QoL and prognosis. However, there are some issues related to exacerbations. They are not easy to define, and the classification of severity is mainly based on subjective criteria. Therefore, it is likely that the assessment of whether or not there is an exacerbation and the degree of severity are influenced by the investigator. Given the nature of our study, these limitations are impossible to avoid. Another aspect is that there are different types of exacerbations not adequately profiled. Even though all exacerbations affect patients in some way, we only included moderate/severe exacerbations in our index for degree of control, given the difficulty to properly diagnose mild exacerbations in a retrospective view. Most of the participating centers had an electronic medical record, which allowed having a reliable information.

### Level of control

Our definition of good control included two groups: optimal and suboptimal. Taking into consideration the fact that the drugs available for COPD treatment do not normalize the clinical situation in a vast majority of patients, we deemed it more realistic and practical to declare “good control” when the values associated with poor prognosis or with high impact on QoL and symptoms were not present, i.e. when mMRC score was < 2, CAT score was < 10 and a maximum of one moderate exacerbation occurred in the previous year. Available evidence suggests that under these circumstances the patient is reasonably controlled, both in terms of symptoms and prognosis [21, 22]. The percentage of patients who reached this degree of good control in our study was 36%, indicating that approximately two-third of patients had a clinical situation that did not meet the minimal requirements. Small differences were observed between PC and RC settings.

### Comparison of our results with previous studies

There are few publications with similar characteristics to our research. A recently conducted study from a database in the United Kingdom proposed three ways to measure the degree of control for a period of 3 months: stability, clinical symptoms or CAT score. Results varied depending on the method used and the level of severity established by the body mass index, airflow obstruction, dyspnea, and exacerbations index (BODEx), which did not achieve a wide acceptance in clinical practice. Among patients with mild/moderate severity (90% of the total), 21.5% were well controlled based on a CAT score < 10; however, only 4.5% were well controlled based on clinical criteria. In the severe/very severe group, none of the patients achieved an adequate control per clinical criteria, while 8.5% of patients achieved adequate control based on a CAT score < 20 [16]. This study is completely different from ours, both in its design and in its objectives. If patients are previously classified according to the degree of severity of the disease, which implies including some of the criteria used to estimate the degree of control, the results can be predicted in a certain way. The aim of our study was to have an overview of the degree of control of patients with COPD in Spain in a sample that was quantitatively representative of the entire geography. The other study, published more recently by the same group, used largely the same parameters that we defined in our study [17]. An interesting finding was that changing the cut-off point of severity in the BODEx index (5 to 3) the percentage of well-controlled patients did not vary significantly, which supports the use of a simple index such as the one we used in our study.

Our results, together with the above findings, reinforce the fact that we are far from obtaining satisfactory rates of good control in COPD patients, regardless of the method used to measure it.

### Level of control PC vs RC

In our study, a slightly higher percentage of patients from RC were well-controlled compared to the PC setting, although no statistical significance was achieved. Even though the most severe patients are usually seen in RC settings, it is likely that other variables, including adherence to treatment, may have influenced this result. It is a field that should be explored in subsequent studies.

### COPD phenotypes and level of control

Patients with exacerbating phenotype were observed to be more often poorly controlled, as was expected based on our definition of good control. Patients with asthma-COPD overlap phenotype showed a degree of control intermediate between exacerbators and non-exacerbators. This was expected as, though they are known to have some exacerbations, patients with this phenotype also show a good response to treatment with inhaled corticosteroids [24]. A limitation to this analysis comes from the fact that each investigator assigned their patients’ phenotype, which may imply differences in criteria.

### Level of control according to treatment

A better degree of control was observed in patients treated with DBD compared with patients treated with a LABA/ICS combination, with greater differences in the RC setting. Obviously, this information has limited value due to the observational nature of our study This finding is not surprising because different studies have shown that the combination of LAMA/LABA is in general the most effective treatment to control the symptoms. Regarding exacerbations, the evidence seems to indicate that the most potent combinations of long-acting bronchodilators outweigh the association of LABA/ICS [15]. Another interesting finding is that in PC 13% of patients with very poor control were treated with a LABA/ICS combination only, even though all the guidelines recommend using LAMA/LABA together with ICS if the patient is a frequent exacerbator. This did not happen in any patient in RC. It is clear that we are still far from using correctly the available treatments.

### Effect of treatment on morning symptoms

Similar results were observed when evaluating the variations in morning symptoms. As expected, patients using a combination of two long-acting bronchodilators achieved a more intense improvement than with any other treatment; however, the clinical relevance of these findings needs to be confirmed in adequate studies. An interesting finding is that there was significant clinical improvement in all stages of GOLD, without differences when applying GOLD 2015 or GOLD 2017. Although this study is not designed for it, these data could support the early use of long-acting bronchodilators even in patients who perceive few symptoms.

## Conclusions

To conclude, we created and tested a simple index to measure the level of control in COPD, built in accordance with the well-accepted guidelines. In our research we observed a very high percentage of COPD patients who do not achieve an adequate control of their disease. We found no significant differences between patients seen in PC and RC settings. The regular use of an index such as the one presented in this work may help improve the disease control and optimize the treatment, as it includes the most relevant variables related to the clinical situation and the risk for the patients. In addition, the index is easily usable in primary care clinics.

## Abbreviations

BODEx: Body mass index, airways obstruction, dyspnea, exacerbations
CAT: COPD assessment test
CI: Confidence interval
COPD: Chronic obstructive pulmonary disease
DBD: Dual bronchodilation
FEV_1_: Forced expiratory volume in 1 s
FVC: Forced vital capacity
GesEPOC: Guía Española de la EPOC
GOLD: Global Initiative for Chronic Obstructive Lung Disease
ICS: Inhaled corticosteroids
LABA: Long-acting β_2_-agonist
LAMA: Long-acting muscarinic antagonist
mMRC: Modified Medical Research Council dyspnea scale
PC: Primary care
QoL: Quality of life
RC: Respiratory care
SD: Standard deviation
